# Nanocellulose–Silver Nanoparticle Nanocomposites for Hydrogel, Aerogel, and Film Development: Antibacterial Properties and Toxicity Evaluation

**DOI:** 10.1021/acsomega.6c02677

**Published:** 2026-06-18

**Authors:** Luiz Gustavo Ribeiro, Yelin Ko, Juan P. Hinestroza, Edison Barbieri, Ana Olivia de Souza

**Affiliations:** † Development and Innovation Laboratory, 196591 Instituto Butantan, São Paulo, São Paulo 05503-900, Brazil; ‡ Department of Surgery, Faculty of Veterinary Medicine and Animal Science, University of São Paulo, São Paulo, São Paulo 05508-220, Brazil; § Department of Human Centered Design, College of Human Ecology, 5922 Cornell University, Ithaca, New York 14853-0001, United States; ∥ Instituto de Pesca, APTA-SAA-Governo Estado São Paulo, Cananeia, São Paulo 05650-905, Brazil

## Abstract

A green and sustainable strategy was employed for the *in situ* formation of silver nanoparticles (AgNPs) within a TEMPO-oxidized cellulose nanofibril (CNF) matrix, using CNF as both a reducing and stabilizing system. AgNPs formation was confirmed by the presence of a surface plasmon resonance band at approximately 420 nm, while DLS indicated colloidal dispersion and transmission electron microscopy (TEM) revealed predominantly spherical nanoparticles, mostly in the 10–20 nm range, associated with the CNF network. The CNF-AgNP nanocomposite dispersion was further processed into hydrogels, films, and aerogels, which were characterized by transmission electron microscopy (TEM), rheology, tensile testing, field-emission scanning electron microscopy (FE-SEM), and thermogravimetric analysis (TGA). Further, the actual silver content in the CNF-AgNP hydrogel was determined by Inductively Coupled Plasma Optical Emission Spectrometry (ICP-OES). The hydrogel exhibited shear-thinning behavior, whereas the films showed a tensile strength of 1.39 MPa and thermal stability up to approximately 100 °C. The CNF-AgNP composites inhibited the growth of *Pseudomonas aeruginosa*, *Staphylococcus aureus*, and *Bacillus subtilis* through disk diffusion assays, while CNF alone showed negligible antibacterial activity. In the Zebrafish (*Danio rerio*) Embryo Toxicity Test (FET Test), end points such as embryo coagulation, lack of somite formation, nondetachment of the tail, and absence of heartbeat were assessed for CNF-AgNPs that induced acute lethality (LC_50_) of 1.115 μM at 96 h, whereas CNF dilutions were nontoxic under the tested conditions. Overall, TEMPO-oxidized CNF enabled the fabrication of multifunctional CNF-AgNP hydrogels, films, and aerogels with antibacterial activity and reduced acute embryotoxicity compared with free silver ions. Further studies addressing silver release kinetics, long-term exposure, and performance under real-world conditions are warranted to substantiate their environmental and biomedical safety.

## Graphical abstract

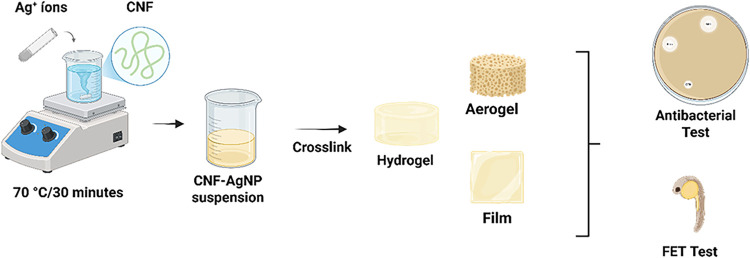

## 1 Introduction

The increasing demand for sustainable and multifunctional materials has driven research into cellulose-based nanocomposites from abundant, renewable, and biodegradable natural resources. Cellulose nanofibrils (CNF) obtained through defibrillation of cellulose at the nanoscale, exhibit remarkable properties such as high mechanical strength, biocompatibility, and the ability to form three-dimensional networks.^1,2^ TEMPO (2,2,6,6-tetramethylpiperidinyl-1-oxyl)-mediated oxidation of CNF introduces carboxylic groups onto its surface. This modification enhances the stability of CNF in aqueous dispersions and facilitates functionalization of the resulting CNF, thereby broadening its potential applications in various fields such as biomedicine, electronics, and packaging.^3,4^

Silver nanoparticles (AgNPs) are well-known due to their antimicrobial, catalytic, and optical properties.^5,6^ However, conventional synthesis methods of AgNPs often involve using toxic reagents, leading to the generation of harmful waste.^7^ In response to these environmental concerns, green synthesis methods for AgNPs have gained significant prominence. These methods utilize reducing and stabilizing agents of natural origin, such as plant extracts, microorganisms, and biopolymers, offering a sustainable alternative to conventional approaches. Further, previous studies have utilized CNF as a reducing and stabilizing agent in AgNP synthesis, employing chemical, photochemical, and biological reduction methods.^8,9^

TEMPO oxidation of CNF increases the density of negative charges on its surface, which can favor interaction with silver ions and facilitate their reduction to AgNPs.^10^ Moreover, the presence of carboxylic groups can increase the stability of AgNPs within the CNF matrix, preventing their aggregation and ensuring a homogeneous distribution.^11^ Considering these properties, the association of CNF and AgNPs in nanocomposites is a promising alternative for developing functional materials with enhanced antimicrobial, mechanical, and thermal properties.^12,13^

In this context, this study reports a faster *in situ* method for the formation of AgNPs in a matrix of TEMPO-oxidized CNF, as reducing and stabilizing agents. In addition, the AgNP-CNF suspension was applied for obtaining hydrogel, aerogel and film, which were characterized by spectroscopy (UV–vis), dynamic light scattering (DLS), Fourier-transform infrared (FTIR), transmission electron microscopy (TEM), Field-Emission scanning electron microscope (FE-SEM), mechanical and rheological properties. The antimicrobial activity of CNF and AgNP-CNF was evaluated on the clinically relevant microorganism, the Gram-negative bacterium *Pseudomonas aeruginosa*, and on the Gram-positive bacteria *Staphylococcus aureus* and *Bacillus subtilis*.

## 2 Materials and Methods

### 2.1 Materials

A water-based dispersion of CNF was obtained from the University of Maine (Orono, ME, USA). The nanofibrils were produced using the TEMPO-mediated oxidation method, with sodium hypochlorite as the terminal oxidant.^14^ The dispersion had a CNF concentration of 0.95 wt % and a carboxylate content of 1.45 mmol per gram of dried CNF. Silver nitrate (AgNO_3_) used as the precursor for AgNPs was purchased from Sigma–Aldrich (≥99.0%, #209139).

### 2.2 Biosynthesis of AgNPs Using TEMPO-Oxidized CNF

AgNPs were synthesized in situ using a modified procedure.^15^ An aqueous solution of AgNO_3_ (4 mM) was mixed with an equal volume of a 0.95 wt % CNF aqueous suspension for final concentration of 2 mM. The mixture was heated to 70 °C under magnetic stirring for 30 min, resulting in a CNF-AgNP suspension, which was then cooled to room temperature (RT) and stored in the dark until further use.

### 2.3 Preparation of Hydrogel, Aerogel and Film

Hydrogel was prepared by transferring 10 mL of the CNF-AgNP suspension into a Petri dish (35 × 10 mm) and allowing the material to gel over 48 h at room temperature under light protection. No external chemical cross-linker was added, therefore, gelation is attributed to physical network formation, driven by nanofibril entanglement and hydrogen bonding as the dispersion ages under quiescent conditions and slight water loss increases the effective solids content, yielding a self-supporting hydrogel. Film was prepared similarly by transferring 5 mL of the CNF-AgNP suspension into a Petri dish (60 × 10 mm), followed by drying for 120 h at RT under light protection. Aerogel were prepared by lyophilizing 20 mL of the CNF-AgNP suspension for 24 h in a freezing dryer, following a prefreezing step at −20 °C for 12 h.

### 2.4 Characterization of the CNF and CNF-AgNP

#### 2.4.1 Spectroscopy Characterization

The absorbance of the CNF and CNF-AgNP suspensions was measured using a UV–vis spectrophotometer (Lambda 35, PerkinElmer) over the 200–800 nm range. FTIR spectra were acquired using a FTIR-4700 spectrometer (JASCO) over the 4000–600 cm^–1^ range, with a resolution of 4 cm^–1^ and 50 scans per sample. The hydrodynamic size and zeta potential of the AgNPs were determined for three replicates of a 10-fold diluted sample using dynamic light scattering (DLS; Zetasizer Nano ZS, Malvern) at 25 °C.

#### 2.4.2 Silver Quantification and Release Kinetics

The actual silver content in the CNF-AgNP hydrogel was determined by Inductively Coupled Plasma Optical Emission Spectrometry (ICP-OES 5100 VDV, Agilent Technologies) as previously described.^16^ For total silver quantification, an aliquot of the hydrogel was digested in sub-boiling distilled nitric acid (HNO_3_, 5% v/v) and diluted with purified water.

Silver release kinetics (leaching) were evaluated by dispersing the CNF-AgNP composite in deionized water at a concentration of 10 μM (the maximum concentration used in toxicity assays). The suspensions were kept at room temperature for 24 and 96 h. At each time point, the supernatant was separated by centrifugation at 5000 rpm for 1 h, followed by filtration through a 0.22 μm membrane to ensure the removal of any suspended fibrils. The filtrate was acidified with 5% HNO_3_ prior to ICP-OES analysis. All measurements were performed in analytical triplicates.

#### 2.4.3 Field-Emission Scanning Electron Microscopy (FE-SEM) and Transmission Electron Microscopy (TEM)

FE-SEM was used to examine the porous structure of the aerogel and the surface of films, and the TEM to characterize the CNF and CNF-AgNP suspensions. For SEM, the samples were mounted on carbon tape, sputter-coated with a carbon layer, and surrounded by silver paste to minimize charging. The Scanning Electron microscope (Zeiss Gemini 500) operated at a voltage of 5 kV. For the TEM analysis, the samples were deposited on TEM grids coated with a carbon film. A drop of diluted aqueous dispersion was added to the samples, which remained on the grid for 5 min. Excess liquid was carefully removed with a filter paper edge, and the grids were air-dried. Then, the samples were negatively stained with uranyl acetate.

#### 2.4.4 Rheological Analysis of the Hydrogel AgNP-CNF

Rheological analysis of the AgNP-CNF hydrogel was conducted using a Haake MARS rheometer (Thermo Scientific) equipped with a parallel plate geometry (25 mm diameter, 1 mm gap). Shear stress sweeps were performed over a shear rate range of 0.1–100 s^–1^ at 25 °C.

#### 2.4.5 Mechanical Properties

The mechanical properties of the films were evaluated using a universal testing machine (Instron 5566, Instron Corporation). Specimens were prepared with a length of 10 mm, a thickness of 0.12 mm, and a width of 7.92 mm. Tensile tests were conducted in triplicate at a crosshead speed of 10 mm/min

#### 2.4.6 Thermogravimetric Analysis (TGA)

The thermal stability of the film and of the aerogel was evaluated using a thermogravimetric analyzer (Q500, TA Instruments) under a nitrogen atmosphere with a flow rate of 50 mL/min. Samples were heated from 25 to 700 °C at a heating rate of 10 °C/min.

### 2.5 Antimicrobial Activity

The antibacterial activity of CNF and AgNP-CNF was evaluated using the disk diffusion method^17^ against the clinically relevant pathogens *P. aeruginosa* (ATCC 9027), *S. aureus* (ATCC 6538), and *B. subtilis* (ATCC 18659). Bacteria were precultured in Luria–Bertani (LB) broth at 37 °C and 180 rpm overnight. The cultures were diluted to a final concentration of 1.0 × 10^6^ CFU/mL, and 100 μL of each suspension was spread onto Petri dishes (90 × 60 mm) containing LB agar. After inoculation, filter paper discs (5 mm diameter) impregnated with 50 μL of AgNP-CNF (2 μM) or CNF suspensions, saline solution (negative control), or gentamicin (10 μg; positive control) were dried for 2 min and then placed onto the plates, in six replicates. Plates were incubated for 24 h, and zones of inhibition were digitally recorded for size analysis. The results were expressed as the mean ± standard deviation.

### 2.6 Zebrafish Embryo Toxicity Test (FET Test)

The zebrafish (*Danio rerio*) embryo toxicity test was performed according to the Organization for Economic Co-operation and Development ((OECD) Test Guideline No. 236)^18^ with slight adaptations. Fertilized embryos at up to 4 h postfertilization (hpf) were selected and randomly distributed into 24-well plates, with one embryo per well containing 2 mL of the test solution.

The CNF-AgNP suspension was tested at 0.1, 0.5, 1, and 5 μM, along with a control group without treatment. As controls, dilutions of the CNF solution (initial concentration 0.95 wt %) were prepared at 1:5, 1:10, 1:20, 1:30, and 1:40 (0.019, 0.048, 0.032, and 0.024 wt %) ratios. Additionally, AgNO_3_ was tested as control at 0.05, 0.1, 0.5, 1, and 5 nM.

Embryos were maintained at 26 ± 1 °C under static conditions without feeding during the 96-h exposure period. End points such as embryo coagulation, lack of somite formation, nondetachment of the tail, and absence of heartbeat were assessed according to OECD guidelines. Each treatment was performed in triplicate with 10 embryos per replicate.

### 2.7 Statistical Analysis

Statistical analyses were performed using GraphPad Prism 10.0 (GraphPad Software, San Diego, CA, USA). One-way ANOVA followed by Dunnett’s multiple comparison test was used to assess differences among groups. Differences compared to control values were considered statistically significant at *P* < 0.05. LC_50_ values were calculated using the trimmed Spearman–Karber method, with a 95% confidence limit.

## 3 Results and Discussion

### 3.1 Characterization of the Preparations by UV–vis and FTIR

The AgNP-CNF suspension exhibited a brown color (Figure 1A), indicative of successful AgNP formation, which was confirmed by UV–vis spectroscopy and FTIR analysis. As shown in Figure 1A, the AgNP-CNF suspension displayed a characteristic surface plasmon resonance (SPR) peak at approximately 420 nm, consistent with previous reports on the synthesis of AgNPs using cellulose-based materials.^19,20^ In contrast, as expected, the CNF suspension was white and did not exhibit a SPR absorption peak (Figure 1A).

**Figure 1:**
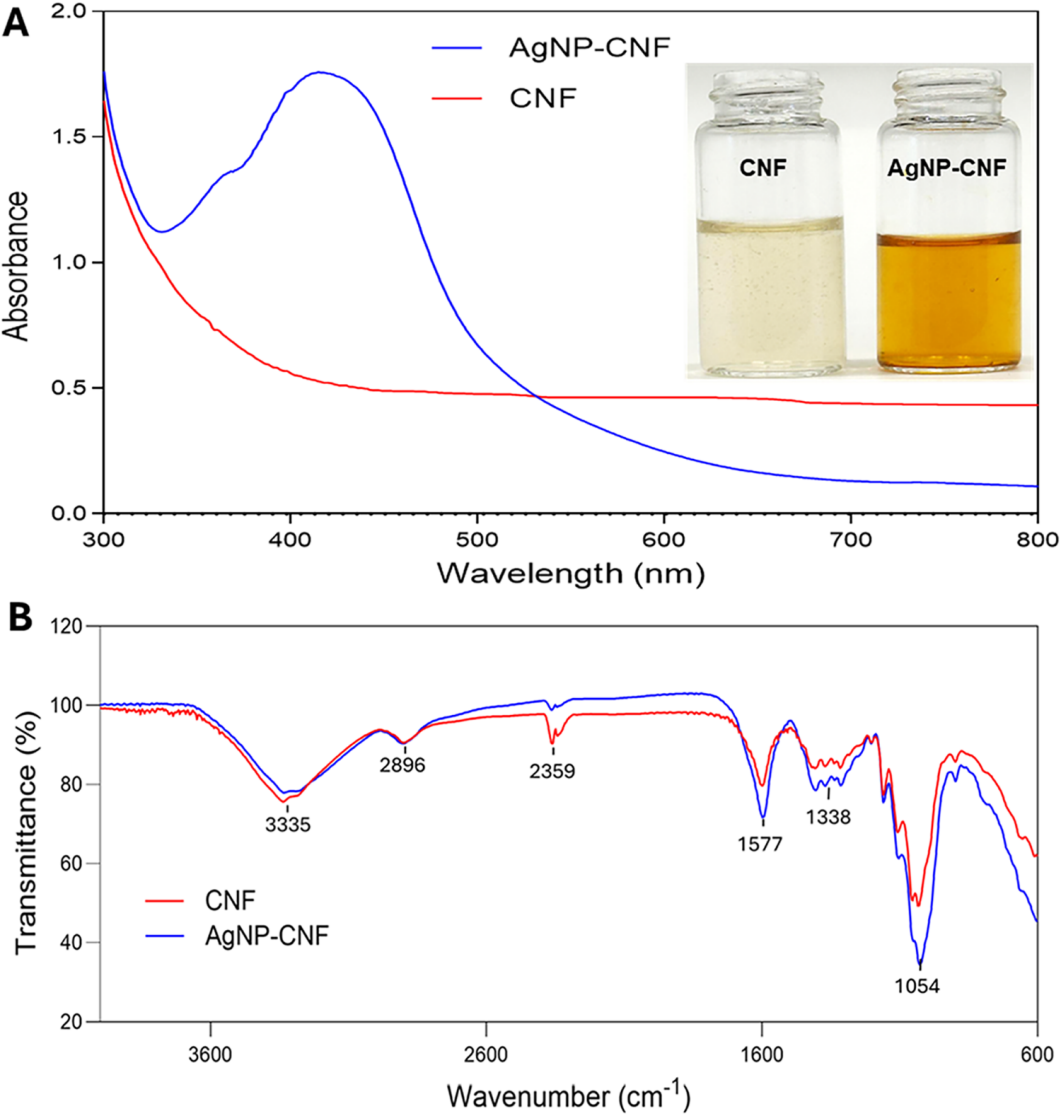
(A) Illustration of the CNF and AgNP-CNF suspensions and UV–vis spectra, showing the characteristic intense peak of AgNPs due to surface plasmon resonance (SPR) around 420 nm. (B) FTIR spectra of CNF and AgNP-CNF.

FTIR spectra were used to discuss functional groups of TEMPO-oxidized CNF and to assess whether the CNF chemical backbone was preserved after AgNP formation. The spectra of CNF and CNF–AgNP (Figure 1B) were dominated by typical cellulose vibrations (broad O–H stretching around 3300 cm^–1^, C–H stretching near 2890 cm^–1^, and C–O/C–O–C vibrations in the 1200–1000 cm^–1^ region). Bands in the fingerprint region associated with TEMPO-derived carboxylate groups (COO^–^ asymmetric and symmetric stretching, typically around ∼1600 and ∼1420 cm^–1^) were retained. Within the spectral resolution, no diagnostic peak shifts that would allow assigning a specific coordination motif were observed; therefore, FTIR was not used here to quantify Ag loading or to “confirm incorporation.” Instead, the formation of AgNPs is supported primarily by the emergence of the SPR band in UV–vis (∼420 nm) and by TEM imaging, while FTIR indicates that the CNF matrix remains chemically intact after the *in situ* reduction.

### 3.2 Silver Loading and Leaching Behavior

ICP-OES analysis confirmed the successful incorporation of silver into the nanocellulose matrix. As expected, the CNF-AgNP hydrogel exhibited a silver concentration of 233 ± 10 mg/L corresponding to 2.1 mM, which provided a precise baseline for calculating the concentrations used in subsequent biological assays.

Regarding stability, the leaching profile demonstrated that the TEMPO–CNF matrix effectively anchors the AgNPs. When the composite was dispersed at 10 μM, the concentration of Ag+ ions released into the medium at 24 and 96 h was 1.20 ± 0.003 mg/L (11 μM) and 1.08 ± 0.04 mg/L (10 μM), respectively. The slight decrease in silver concentration over time may suggest a readsorption equilibrium between the released ions and the carboxylated groups of the CNF surface.

### 3.3 Morphology of AgNP-CNF Composites

#### 3.3.1 AgNP-CNF Hydrogel

Figure 2A shows a digital photograph of the self-supporting CNF-AgNP hydrogel obtained after aging the CNF-AgNP dispersion. TEM grids were prepared by drop-casting diluted dispersions onto carbon-coated grids; consequently, the micrographs in Figure 2B–D provide information on the nanoscale morphology of CNF fibrils and AgNPs in the hydrogel precursor rather than the fully hydrated three-dimensional hydrogel architecture.

**Figure 2:**
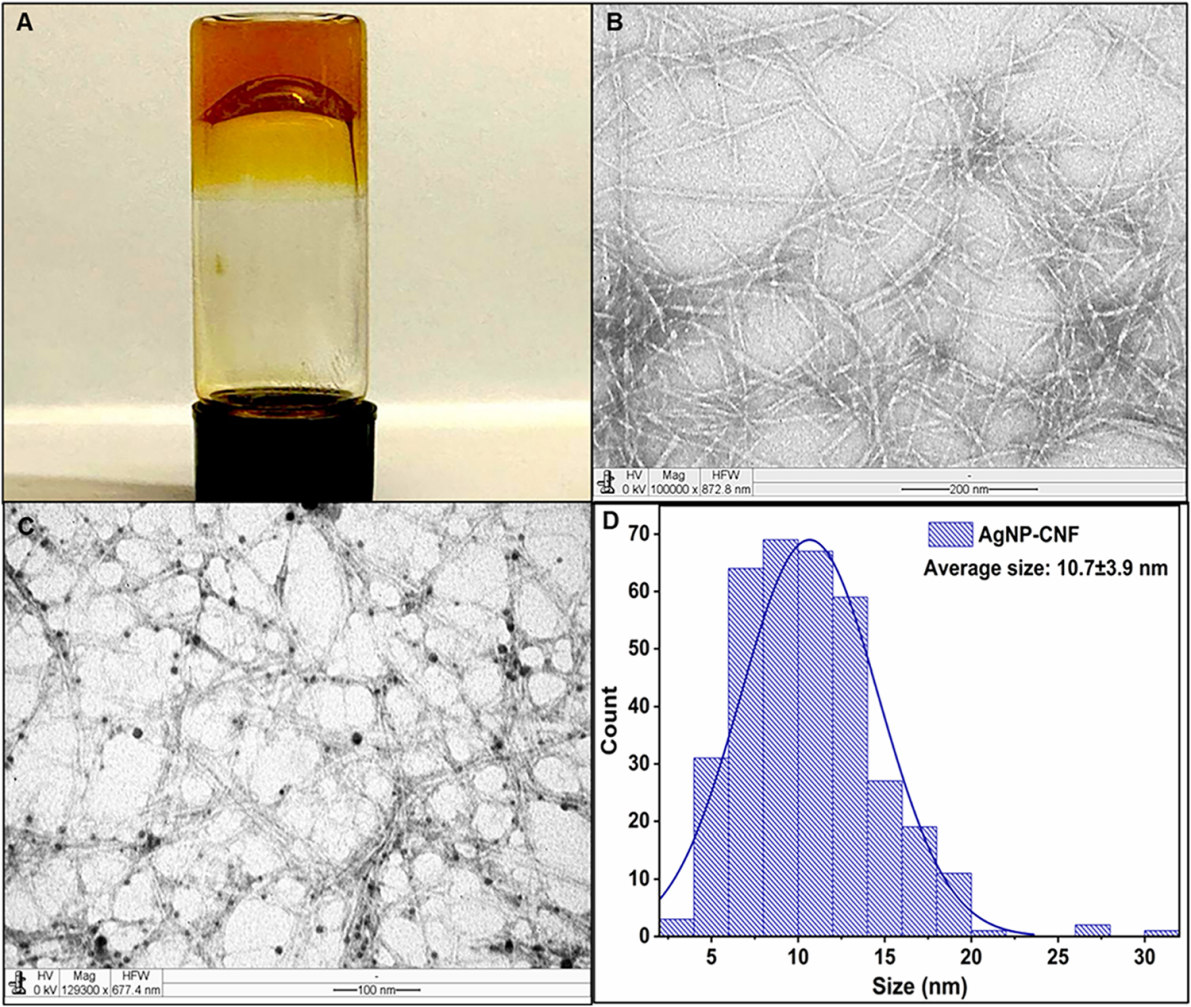
(A) Digital photography of the self-supporting CNF-AgNP hydrogel. Transmission electron microscopy (TEM) micrographs obtained from (B) drop-cast, diluted CNF and (C) CNF-AgNP dispersions (hydrogel precursors) on carbon-coated grids. (D) Particle diameter distribution histogram derived from TEM analysis. The images support AgNP morphology and association with CNF fibrils, but do not directly represent the hydrated bulk hydrogel network. (B, C—magnification of 100,000× and 129,300× respectively). Scale bars: 100 and 200 nm.

As observed in Figure 2C, the AgNPs were predominantly spherical and frequently localized in proximity to CNF fibrils. A particle diameter histogram (Figure 2D), quantified from TEM micrographs (*n* = 350 particles from 25 independent images/fields of view), yielded an average diameter of 10 ± 4 nm. The NPs exhibited a good and uniform distribution within the CNF-AgNP hydrogel, consistent with previous reports.^21^ This uniform distribution is attributed to favorable interactions between the negatively charged carboxylate groups on the CNF surface and the positively charged silver ions, which prevent aggregation and promote the anchoring of AgNPs onto the CNF matrix.^20^ Figure 2B displays the CNF suspension, while Figure 2C highlights the nanofibrous structure of the composite, demonstrating the homogeneous dispersion of AgNPs throughout the polymeric matrix. The association between AgNPs and TEMPO-oxidized CNF is consistent with adsorption/coordination at oxidized surface functionalities (e.g., carboxylate groups) and with electrostatic stabilization of the dispersion, which may contribute to limiting macroscopic aggregation during gel formation.

#### 3.3.2 AgNP-CNF Aerogel

The morphology of the aerogel was investigated using FE-SEM. The micrographs (Figure 3A–C) show a continuous CNF-based network with a relatively compact/lamellar morphology formed during freeze-drying, with micron-scale pores and interconnected domains. Under the imaging conditions employed, individual AgNPs could not be unambiguously resolved by SEM; therefore, Figure 3 is discussed primarily in terms of aerogel architecture rather than nanoparticle localization. Nevertheless, the absence of large, high-contrast particulate agglomerates at the microscale is consistent with the colloidal stability observed for the CNF–AgNP dispersion prior to drying. Evidence for AgNP formation and nanoscale morphology is provided by UV–vis (SPR band) and TEM (Section 3.1 and 3.2). This homogeneous dispersion is attributed to the stabilizing effect of the CNF, which acts as a support matrix preventing nanoparticle agglomeration.^22^

**Figure 3:**
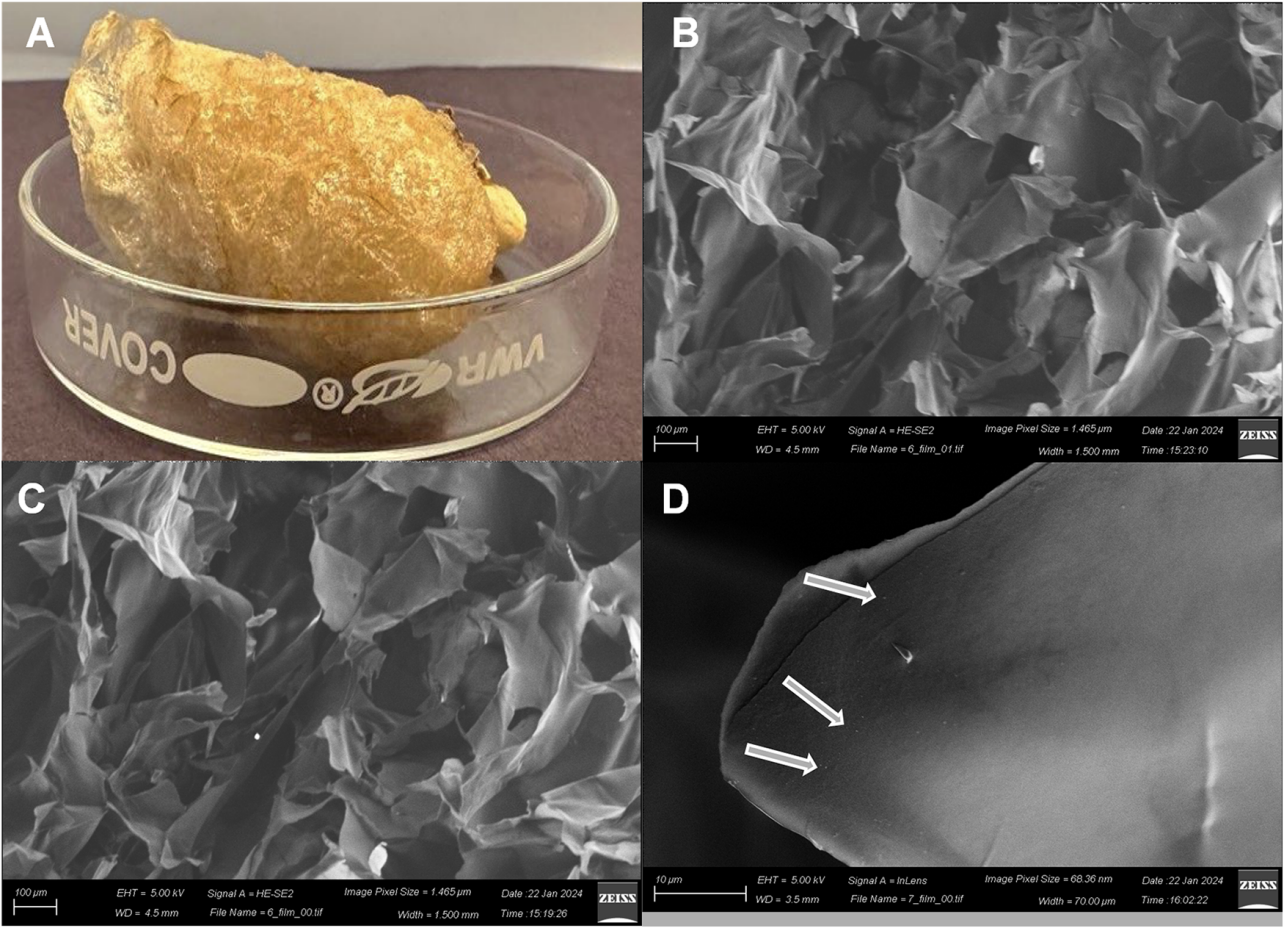
(A) Digital image of the CNF-AgNP aerogel in a Petri dish. (B–D) Field-emission scanning electron microscopy (FE-SEM) micrographs of the aerogel. (D) Arrows indicate regions of higher contrast within the CNF network; however, elemental mapping was not performed, and nanoparticle presence is supported primarily by UV–vis/TEM Scales bar:10 and 100 μm.

#### 3.3.3 AgNP-CNF Film

The morphology of the film produced from the CNF-AgNP suspension is shown in Figure 4A. TEM micrographs (Figure 4B–D) reveal a distributed surface with AgNPs evenly dispersed throughout the CNF matrix. The dispersion is facilitated by the CNF, which acts as a stabilizing agent, enabling the formation of spherical and uniformly distributed AgNPs. TEM analysis of the film (Figure 4B–D) indicated predominantly spherical nanoparticles. Particle size was quantified from TEM micrographs (*n* = 250 particles), giving an average diameter of **∼**16 nm (Figure 4D), consistent with the nanoscale morphology observed.

**Figure 4:**
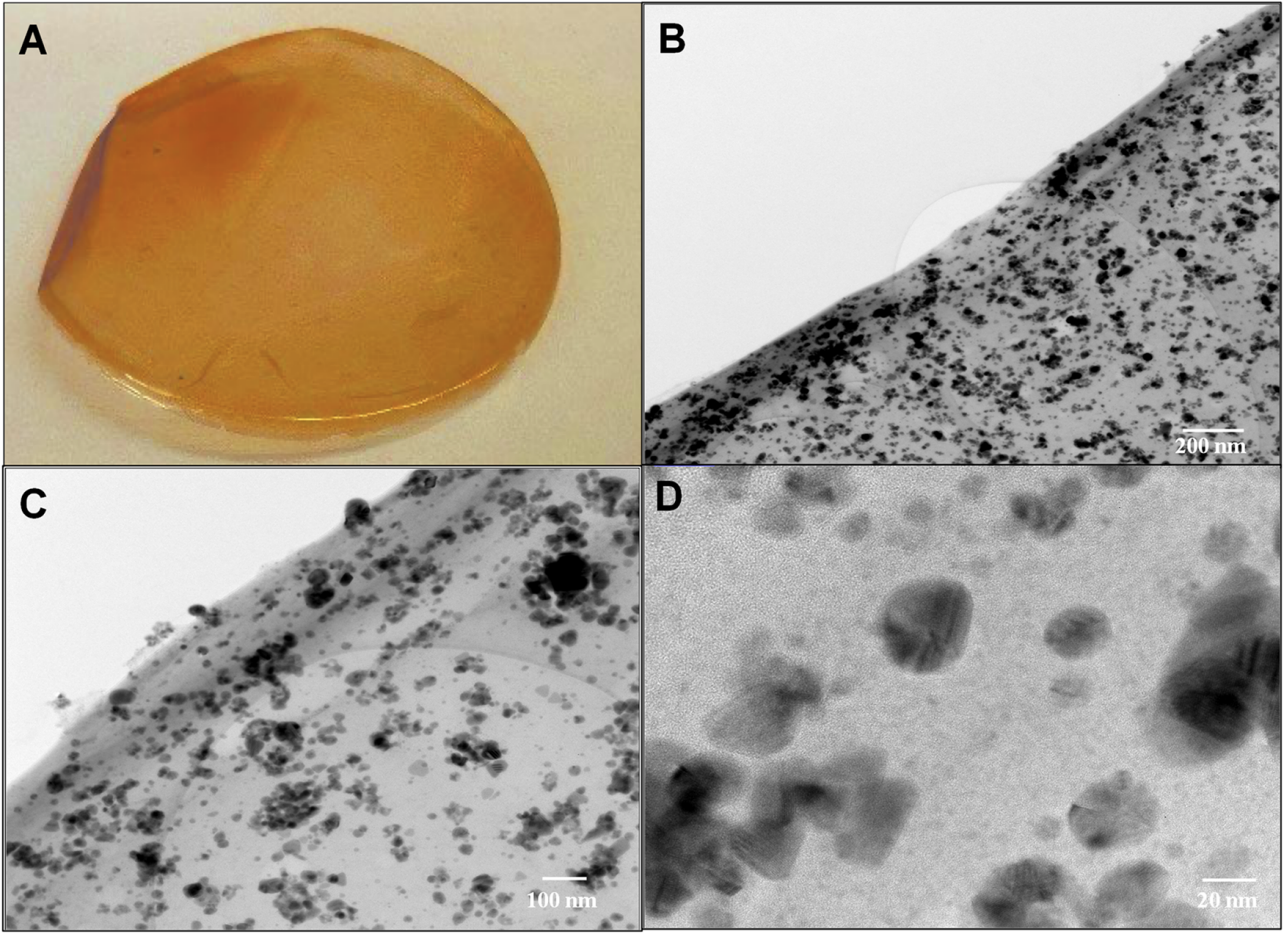
(A) Digital image of the AgNP-CNF film. (B–D) Transmission electron microscopy (TEM) micrographs of the AgNP-CNF film at different scale bars (200, 100, and 20 nm). The AgNPs are spherical and exhibit a size distribution of 20 ± 10 nm.

### 3.4 Rheological Properties of the AgNP-CNF Hydrogel

The graphs presented in Figure 5 illustrate the rheological behavior of the AgNP-CNF hydrogel, showing the relationship between shear stress and shear rate (Figure 5A) and between viscosity and shear rate (Figure 5B). The shear stress versus shear rate curve displays a nonlinear profile, indicating that the AgNP-CNF hydrogel behaves as a non-Newtonian fluid. Initially, the curve exhibits a steeper slope, followed by a region with a lower slope, suggesting a decrease in viscosity with increasing shear rate. This behavior, known as shear thinning or pseudoplasticity, is characteristic of materials whose viscosity decreases under shear. The viscosity versus shear rate curve further confirms this shear-thinning behavior, demonstrating a progressive reduction in viscosity as the shear rate increases. This phenomenon can be attributed to the alignment of CNF in the direction of flow, which reduces entanglement and interfibrillar interactions, thereby facilitating fiber sliding and decreasing flow resistance.

**Figure 5:**
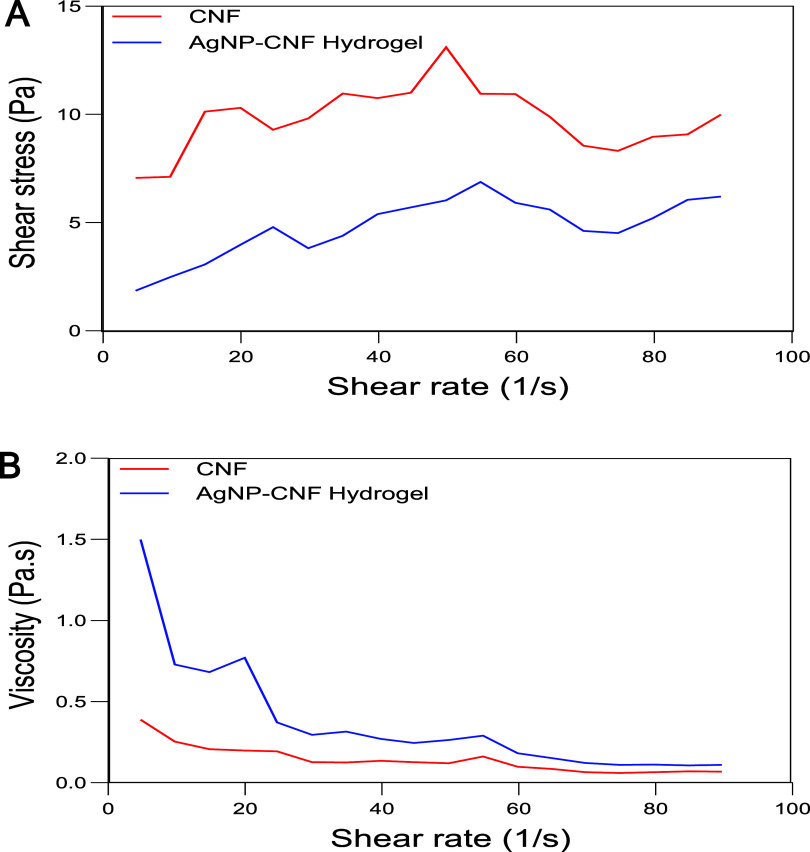
Representation of the rheological behavior of the AgNP-CNF hydrogel. (A) shear stress as a function of shear rate, and (B) viscosity as a function of shear rate.

### 3.5 Mechanical Properties and Thermogravimetric Analysis of the AgNP-CNF Film

Mechanical properties are critical for the practical application of materials. Therefore, a tensile test was performed to evaluate the mechanical characteristics of the CNF-AgNP film. Figure 6A presents the stress–strain curve of the sample. The film exhibited brittle behavior with limited flexibility, fracturing without significant plastic deformation.

**Figure 6:**
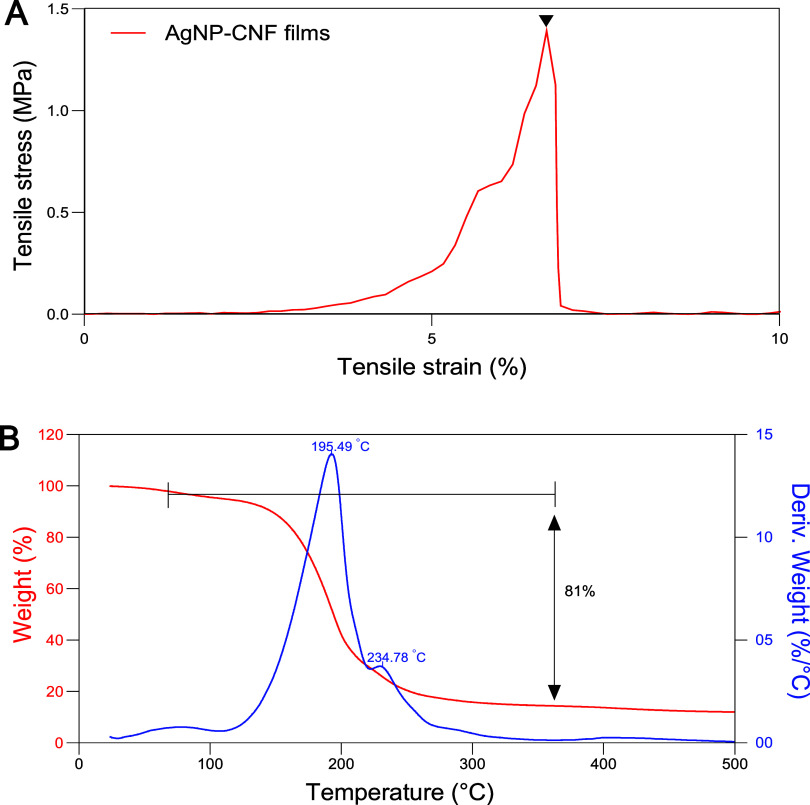
(A) Representation of the stress–strain curve of the AgNP-CNF film, where the triangle indicates the tensile strain at break, and of the (B) thermogravimetric analysis. The blue line represents the DTG curve, and the red line represents the lost-weight TGA curve.

The film showed a rupture stress of 1.39 MPa, demonstrating relatively high tensile strength. However, the rupture strain was only 6.65%, indicating low extensibility before failure. The rupture load applied to the film was 1.3225 N. These mechanical characteristics have important implications for practical applications, particularly in scenarios requiring high strength combined with minimal dimensional changes under stress.

The TGA curve of the CNF-AgNP film (Figure 6B) exhibits an initial mass loss between 80 and 100 °C, likely associated with the evaporation of residual water. The main degradation stage occurs between 100 and 300 °C, characterized by a sharp mass loss corresponding to the thermal decomposition of volatile components and cellulose within the nanofibers. The DTG curve displays two prominent peaks at approximately 195 and 244 °C, indicating the highest degradation rates. The presence of two peaks suggests a two-step decomposition process: the first peak (195 °C) may be attributed to the decomposition of surface functional groups or low molecular weight compounds, while the second peak (244 °C) corresponds to the decomposition of cellulose, the primary component of the film.^23^

Overall, the TGA results reveal that the CNF-AgNP film exhibits good thermal stability up to approximately 100 °C. Beyond this temperature, the decomposition of volatile components initiates, followed by cellulose degradation at higher temperatures. The incorporation of AgNPs does not significantly affect the onset degradation temperature but may influence the degradation rate, as evidenced by the features observed in the DTG curve.

### 3.6 Antibacterial Activity of the AgNP-CNF Suspension

Figure 7 illustrates the antibacterial activity of the AgNP-CNF suspension, as evidenced by the formation of inhibition zones around the discs impregnated with AgNP-CNF against *S. aureus*, *P. aeruginosa*, and *B. subtilis*. The AgNP-CNF suspension induced inhibition zones of 12 ± 5 mm, 10.25 ± 5 mm and 12.25 ± 6 mm for *S. aureus P. aeruginosa* and *B. subtilis*, respectively. For comparison, gentamicin produced inhibition zones of 16.6 ± 4 mm, 18 ± 3 mm, and 17.5 ± 4 mm, respectively. These results highlight the significant antibacterial activity achieved through the incorporation of AgNPs into the CNF matrix. As expected, the saline and CNF solutions did not inhibit pathogen growth, except for CNF, which produced a small inhibition zone of 0.9 ± 1 mm against *P. aeruginosa*.

**Figure 7:**
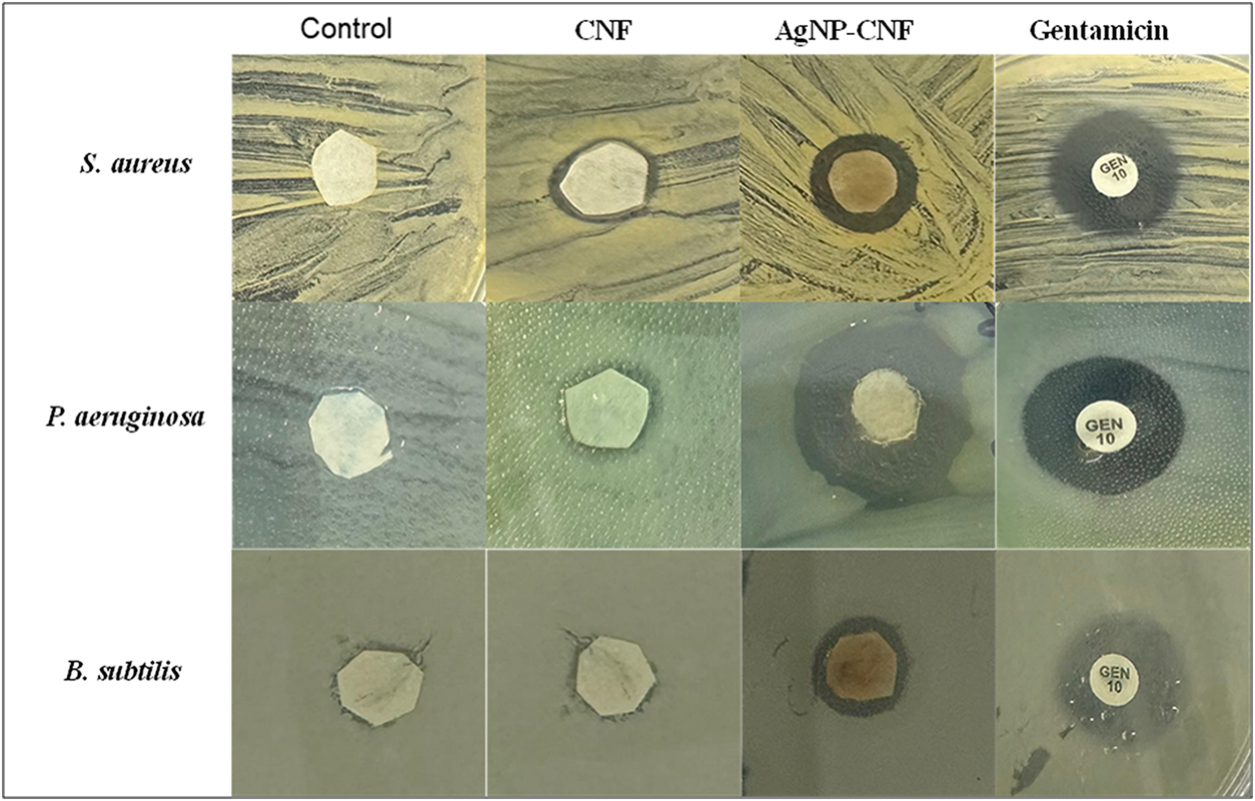
Antibacterial activity of the AgNP-CNF suspension represented by the inhibition zones size against *S. aureus*, *P. aeruginosa*, and *B. subtilis*. Saline solution (control), gentamicin, and CNF were used as controls.

Gentamicin produced larger inhibition zones than CNF–AgNP under the same conditions, confirming bacterial susceptibility and providing a qualitative benchmark for assay performance. Importantly, disk diffusion reflects not only intrinsic antimicrobial potency but also diffusion through agar and interactions with the CNF matrix, so direct potency ranking versus gentamicin should be interpreted cautiously. MIC and plate-count/time-kill assays would be valuable for quantitative comparison but are beyond the scope of the present data set.

### 3.7 Zebrafish Embryo Toxicity Test

The Fish Embryo Toxicity (FET) test allowed a comprehensive evaluation of the acute toxicity of the CNF-AgNP suspension and the pure CNF matrix. The 96-h LC_50_ for the CNF-AgNP suspension was 1.115 μM (95% CI: 0.668–1.862 μM), whereas for the AgNO_3_ was 0.1228 μM (95% CI: 0.03861–0.3905 μM). These findings indicate that the nanocomposite is approximately 10 times less toxic than free silver ions, rather than the initial estimation. No mortality was observed in embryos exposed to CNF at 0.019 and 0.024 wt % (corresponding to dilutions of 1:5 to 1:40), confirming the inherent biocompatibility and absence of toxic effects from the nanocellulose matrix alone.

To correlate the observed biological effects to the actual silver concentration, ICP-OES analysis was performed. The results indicate that, although silver was detected in the exposure medium, its bioavailability and associated toxicity were substantially lower than those observed for AgNO_3_. The reduction in silver concentration from 11 μM at 24 h to 10 μM at 96 h suggests a dynamic adsorption/readsorption equilibrium, likely mediated by carboxylate groups present on the surface of TEMPO-oxidized CNF. This interaction may regulate the release of Ag+ ions into the medium, thereby attenuating its toxicological mechanisms commonly, including oxidative stress, mitochondrial dysfunction, and disruption of ionic homeostasis.^27,28^

In contrast to AgNO_3_, the CNF-AgNP nanocomposite appears to reduce acute toxicity by physically and chemically stabilizing AgNPs within the polymeric CNF network.^29,30^ Overall, these findings suggest that CNF-AgNP composites may represent a safer alternative to free Ag+ ions, particularly in applications where controlled release is desirable. The stabilizing role of the CNF matrix may help reduce acute embryotoxicity while maintaining the functional potential of silver-based nanomaterials for applications such as in biomedical devices, food packaging, and water treatment systems.^23–26^

## 4 Conclusions

This study demonstrated that the incorporation of AgNPs into a TEMPO-oxidized CNF matrix through a green synthesis approach resulted in stable nanocomposites with an uniform nanoparticle distribution. The CNF-AgNP materials successfully combined antibacterial functionality with favorable physical properties, including shear-thinning behavior in hydrogels and thermal stability up to 100 °C in films and aerogels. The CNF-AgNP composites showed antibacterial effectiveness against the clinically relevant bacteria, *S. aureus*, *P. aeruginosa*, and *B. subtilis*.

Furthermore, CNF-AgNPs exhibited lower acute toxicity to zebrafish embryos than free AgNO_3_, with an approximately 9-fold higher 96-h LC50 value, while the CNF matrix alone showed no acute toxicity under the tested conditions. These results indicate that embedding AgNPs within a CNF matrix can reduce the risks typically associated with silver-based antimicrobials by modulating silver availability and improving the environmental and biological safety profile of the material. Therefore, CNF-AgNP nanocomposites emerge as promising sustainable materials for applications requiring antimicrobial properties, structural integrity, and biocompatibility. Future studies should further investigate Ag+ ions release kinetics, long-term stability, antimicrobial durability, and the safety of these composites under real-world biomedical and environmental conditions. Therefore, CNF–AgNP nanocomposites emerge as promising sustainable materials for applications requiring antimicrobial activity.
